# Acetylation of microtubule-binding PinX1 orchestrates ribosome biogenesis to nutrient starvation *via* the RNA polymerase I preinitiation complex

**DOI:** 10.1016/j.jbc.2025.110465

**Published:** 2025-07-08

**Authors:** Gang Lu, Liqian Yang, Qinxin Zhang, Shiqiang Zhang, Yuqi Xiong, Tong Li, Yanli Zhang, Minghui Liu, Yu Zhang, Jiaxing Wu, Qiaoyou Weng, Xiaoyun Liu, Jiansong Ji, Haiying Wang, Jianyuan Luo

**Affiliations:** 1 Department of Medical Genetics, Center for Medical Genetics, School of Basic Medical Sciences, Peking University Health Science Center, Beijing, China; 2 Beijing Key Laboratory of Protein Posttranslational Modifications and Cell Function, Department of Biochemistry and Molecular Biology, School of Basic Medical Sciences, Peking University Health Science Center, Beijing, China; 3 Department of Laboratory Medicine, Peking University Shenzhen Hospital, Shenzhen, Guangdong, China; 4 Zhejiang Key Laboratory of Imaging and Interventional Medicine, Department of Radiology, Lishui Central Hospital, The Fifth Affiliated Hospital of Wenzhou Medical University, Lishui, China; 5 Department of Microbiology and Infectious Disease Center, NHC Key Laboratory of Medical Immunology, School of Basic Medical Sciences, Peking University Health Science Center, Beijing, China; 6 Medical Innovation Center (Taizhou) of Peking University, Taizhou, China

**Keywords:** PinX1, post-translational modification (PTM), POLR1G, ribosome biogenesis

## Abstract

The shutdown of ribosome biogenesis is one of the sophisticated strategies for cells to save energy in response to nutrient starvation. However, the mechanism orchestrating ribosome biogenesis with cellular nutrition status remains unclear. Here, we identified the role of PIN2/TRF1-interacting telomerase inhibitor 1 (PinX1) in regulating ribosome biogenesis. PinX1 is highly expressed in colorectal cancers (CRC). Depletion of PinX1 impairs rDNA transcription, compromises ribosome biogenesis and inhibits tumor cells proliferation. Mechanically, associated with UBTF, PinX1 directly binds to RNA polymerase I subunit G (POLR1G) which is required for the assembly of RNA polymerase I preinitiation complex (PIC). Upon nutrient starvation, PinX1 is acetylated at K43, K133, K140, K149, K190, K222, which hinders its binding to POLR1G leading to disassembly of PIC. Collectively, our findings uncover a novel role of PinX1 and its acetylation, fine-tuning nucleolar transcription to stress signaling.

Stimulated by environmental changes, cells employ a multitude of strategies to obtain adaptation and survival. Different stress conditions may trigger various cellular responses which result in temporary adaptation to stressors or cell death. Under mild nutrient starvation, cells survive with a minimum level of energy consumption through modulating metabolic rearrangements, such as inhibiting mammalian target of rapamycin (mTOR) signaling, inducing autophagy, or suppressing ribosome biogenesis (1, 2, 3). As a major integrator at the crossroads of protein/sugar/fat catabolism, acetyl-coenzyme A (Acetyl-CoA) in the nucleus and cytoplasm is rapidly depleted under nutrient starvation (4, 5). Consequently, deprivation of acetyl groups donor dramatically impacted acetyltransferases on their catalytic activity, which leads to a reduction of the acetylation of most proteins (5). Therefore, we hypothesized that in this acute condition, the retention or even upregulation of acetylation of proteins could be critical for cell survival. Utilizing the TMT-labeling proteomics approach and LC-MS/MS, we previously captured 58 hyperacetylated proteins compared with the overall hypoacetylation status under starvation conditions in HCT116 cells (6). To further understand the relationship between nutrient starvation and acetylation, we investigated the impact of PIN2/TERF1-interacting telomerase inhibitor 1 (PinX1), which is among the top-scoring hits.

PinX1 was characterized as a potent telomerase inhibitor (7) and a microtubule-binding protein (8). It is localized both in the nucleoplasm through its N-terminal and nucleolus through its C-terminal (9). In addition, the telomerase inhibitory domain (TID), located at the C terminus of PinX1, binds the telomerase catalytic subunit hTERT and directly inhibits its activity (7, 10, 11). Overexpression of PinX1 shows profound inhibition of telomerase activity in HT1080 (7), HeLa (12), NIH3T3 (13), and human colorectal carcinoma cells (14). However, PinX1 is not solely a negative regulator of telomerase (15). Accumulating studies have found that silencing PinX1 compromises telomere length maintenance as well as tumorigenicity in HT1080 (16), HepG2 (16, 17), and osteosarcoma cells (18). Complicated functions of PinX1 endow it with a versatile role in cancers, namely inhibiting malignancy in gastric (19), breast (20, 21) and renal cancer cells (22), or promoting proliferation in glioblastoma (23) and prostate cancer cells (24). Tumor type specificity and posttranslational modification of PinX1 may elucidate its duality in regulating cell growth. Nevertheless, the posttranslational modification of PinX1 has yet to be fully investigated. Plk1-mediated phosphorylation of PinX1 was solely identified to regulate its stability (25). The role of PinX1, as well as its acetylation we newly found in response to nutrient starvation, remains elusive.

Enhanced ribosome biogenesis is a common feature of cancer cells, contributing to protein synthesis and cell growth (26). Ribosome biogenesis is a multi-step process initiated by the rate-limiting transcription of ribosomal DNA (rDNA) in the nucleolus. The rDNA harbors about 400 units composed of a precursor rRNA (pre-rRNA) coding region and a long intergenic spacer, which are transcribed by nucleolar RNA polymerase I (Pol I). Synergistic binding of an upstream binding transcription factor (UBTF) and the selectivity factor 1 (SL1) to the rDNA promoter is fundamental to the recruitment of Pol I and assembly of the Pol I preinitiation complex (27, 28), which consumes a lot of cellular energy (29). The rDNA transcription can be fine-tuned in response to environmental cues, including the availability of growth factors and nutrients. Many cellular nutrient and energy-sensing pathways, such as AMP-activated protein kinase (AMPK) signaling, mTOR signaling, and autophagy, have been utilized by cells to link the availability of nutrients and energy to rDNA transcription (29, 30, 31).

mammalian Pol I is composed of 14 subunits: 11 core Pol I subunits and three polymerase-associated factors, namely, RRN3 and a heterodimer of POLR1E/POLR1G (32). Previous studies demonstrated that POLR1G interacting with POLR1E was essential for rDNA transcription and cell proliferation (33, 34, 35, 36). POLR1E fails to recruit Pol I to rDNA in glucose deprivation conditions (37). Upon energy deprivation, rRNA biogenesis is inhibited for the sake of saving energy; in the meantime, autophagy is activated to produce energy and facilitate cell survival (1). However, the molecular mechanisms orchestrating ribosome biogenesis with cellular nutrition status are not fully understood.

In this study, we revealed that PinX1 is highly expressed in colorectal cancers (CRC) and promotes tumor cell proliferation. We identified that PinX1 harbors acetylation, which is dynamically regulated by acetyltransferase CBP and deacetylase HDAC3/HDAC10. PinX1 is required in rDNA transcription through recruiting POLR1G to UBTF. In response to nutrient starvation, PinX1 was acetylated and hyperacetylated. PinX1 repressed its interaction with POLR1G, which led to a decrease in assembly of the Pol I preinitiation complex. Both depletion and hyperacetylation of PinX1 showed compromised ribosome biogenesis and cell growth. Our results suggest a novel role of PinX1 in rDNA transcription and an important regulatory modification of PinX1 in response to nutrient starvation.

## Results

### PinX1 is highly expressed in CRC and promotes CRC proliferation

**Figure 1:**
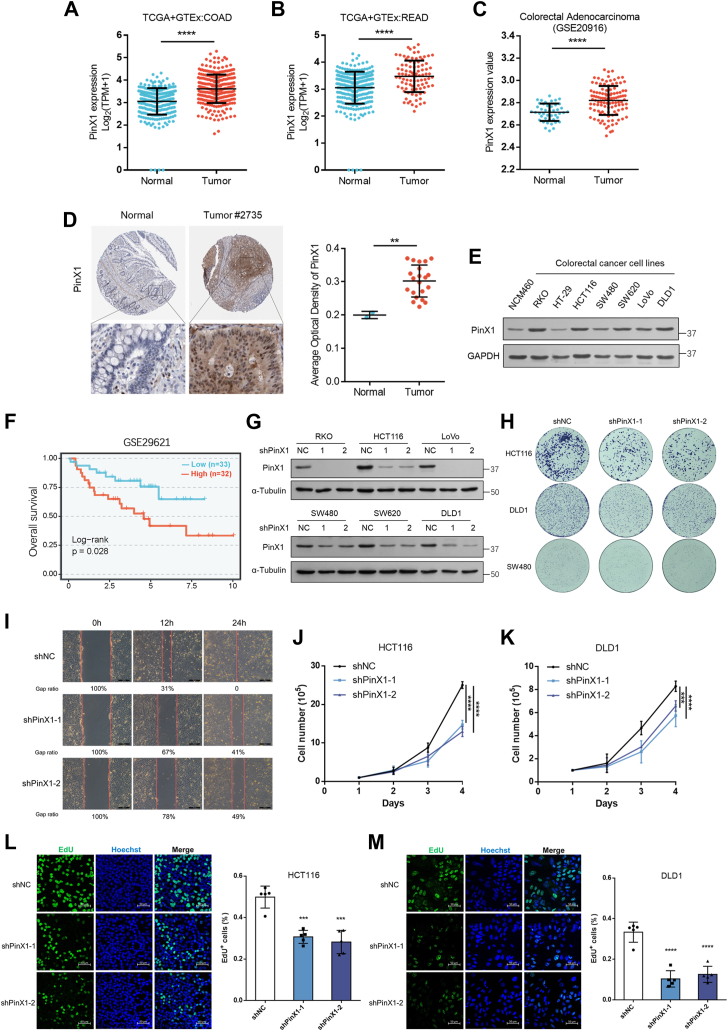
**PinX1 is overexpressed in CRC cells and promotes cell proliferation.***A* and *B*, Scatter plots of PinX1 expression levels of normal tissues and tumor tissues in colon adenocarcinoma (COAD, *left*) and rectum adenocarcinoma (READ, *right*) from TCGA and GTEx database. *C*, Scatter plots of PinX1 expression values of normal tissues and tumor tissues in colorectal adenocarcinoma from GEO database (GSE20916). Data were analyzed using two-tailed unpaired Student’s *t* test. Error bars indicate the mean ± SD. ∗∗∗∗*p* < 0.0001. *D*, IHC staining of PinX1 in normal colon (n = 2) and CRC tissues (n = 21) from HPA database. Average optical density data were analyzed using Mann–Whitney *U* test. Error bars indicate the mean ± SD. ∗∗*p* < 0.01. *E*, Western blot analysis of PinX1 protein levels comparing one normal colon epithelial cell line with seven CRC cell lines. *F*, Kaplan–Meier plots of colon cancer patients stratified by PinX1 mRNA levels from BEST web application, based on the GEO database (GSE29621). *G*, Validation of the knock-down efficiency of PinX1 in CRC cells infected with lentivirus carrying shRNA or PLKO.1 vector using western blotting. *H*, wild-type and PinX1 knockdown cells as indicated were seeded into 6-well plates at 2 × 10^3^ per well, and the cell colonies were stained with crystal violet after 7 days. *I*, wound-healing assays were performed in DLD1 cells (WT, shPinX1-1 or shPinX1-2). Images were captured at times 0, 12, and 24 h. Scale bars = 100 μm. *J* and *K*, wild-type and PinX1 knockdown cells were seeded into 6-well plates at 10^5^ cells per well, and the cell numbers were counted every day. *L* and *M*, the representative image of EdU incorporation assay in wild-type and PinX1 knockdown HCT116 and DLD1 cells (*left*), Scale bars = 50 μm. The percentage of cells positively stained with EdU was quantified (*right*) using one-way ANOVA. Error bars indicate the mean ± SD (n = 5). ∗∗∗*p* < 0.001, ∗∗∗∗*p* < 0.0001.

To investigate the role of PinX1 in cancers, we examined the expression levels of PinX1 in The Cancer Genome Atlas (TCGA) and GTEx database and found PinX1 was elevated both in colon adenocarcinoma (COAD) and rectum adenocarcinoma (READ) compared with the normal tissues (Fig. 1, *A* and *B*). A similar observation was obtained from a GEO database of colorectal adenocarcinoma (Fig. 1*C*). The immunohistochemical staining data of colorectal normal tissues and cancer tissues from the Human Protein Atlas (HPA) database were analyzed. PinX1 was upregulated in CRC tumor tissues compared with non-tumorous colorectal tissues (Fig. 1*D*). These results were further confirmed by the Western blot analysis through assessing the endogenous expression levels of PinX1 in 7 different colon cancer cell lines and one normal human colon mucosal epithelial cell line, NCM460. We found that PinX1 was elevated in most colon cancer cells compared with NCM460 cells (Fig. 1*E*). In addition, patients with higher PinX1 expression exhibited a poor overall survival (Fig. 1*F*), based on the BEST web application (38) and the GEO database (GSE29621). Together, these results indicate that PinX1 expression levels are increased and correlate with a poor prognosis in CRC patients.

To determine whether PinX1 promotes CRC cell proliferation, we generated PinX1 knockdown cell lines using short hairpin RNAs (shRNAs) in RKO, HCT116, LoVo, SW480, SW620 and DLD1 cells, which exhibit relatively high PinX1 expression (Fig. 1*G*) and observed that PinX1 deficiency significantly impaired the colony formation ability (Fig. 1*H*), cell migration (Figs. 1*I* and S1*A*) and cell proliferation rates (Figs. 1, *J* and *K* and S1*B*). Besides, we also found that PinX1 deficiency impaired the proliferative capacity of HCT116, DLD1, SW480, SW620, and RKO cells through the 5-ethynyl-2-deoxyuridine (EdU) incorporation assay (Figs. 1, *L* and *M*, and S1, *C*–*E*). These results demonstrated that high PinX1 expression is crucial to CRC cell proliferation.

### PinX1 is required for rDNA transcription and ribosome biogenesis

**Figure 2:**
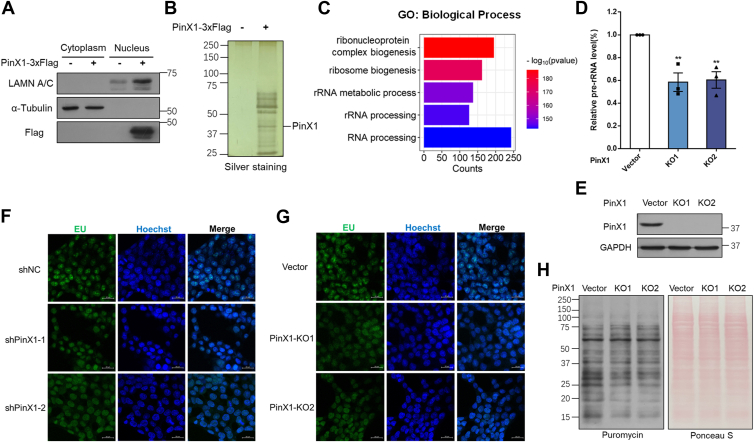
**PinX1 is required for rDNA transcription and ribosome biogenesis.***A*, validation of the nuclear and cytoplasmic fraction by western blotting. *B*, nuclear fraction was immunopurified with anti-Flag agarose beads. The elution was then subjected to SDS–PAGE and silver staining. *C*, the bar graph shows the enrichment analysis of PinX1-associated proteins in the terms of GO biological process. *D*, cellular 47S rRNA (pre-rRNA) levels were measured as indicated by real-time PCR and normalized to ACTB mRNA. Error bars indicate mean ± SD of triplicate experiments. ∗∗*p* < 0.01. *E*, validation of the knockout efficiency of PinX1 in HCT116 using western blotting. *F* and *G*, cells were pulsed with 5-ethynyl uridine (EU) for 10 min, and *de novo* synthesized RNA (*green*) was visualized as described against a Hoechst (*blue*) background. Scale bar = 20 μm. *H*, PinX1 wild type and PinX1-KO HCT116 cells were pulse treated with puromycin (10 mg/ml, 30 min) before harvest. Lysates were analyzed by Western blotting. Ponceau S staining was used as the loading control.

To gain insight into the function of PinX1 at the nucleus, we overexpressed a 3 × Flag-tagged PinX1 in HEK293T cells. These cells were then subjected to nuclear fraction (Figs. 2*A* and S2*A*) and co-immunoprecipitation (co-IP). All the PinX1 proteins are located in the nucleus and we then purified PinX1-associated proteins, which were visualized by silver staining (Fig. 2*B*) and identified by mass spectrometry. To determine the role of these PinX1-associated proteins, we performed a cluster analysis, which indicated that these proteins were highly related to ribosome biogenesis (Fig. 2*C*). Since rDNA transcription is the first and rate-limiting step in ribosome biogenesis, the mass spectrometry result prompted us to investigate a possible role of PinX1 in rDNA transcription. We generated PinX1 knockout HCT116 cell lines using CRISPR-Cas9 and monitored pre-rRNA synthesis by quantitative real-time PCR analysis. Depletion of PinX1 decreased the pre-rRNA levels (Fig. 2, *D* and *E*). Moreover, knockdown of PinX1 by siRNAs decreased pre-rRNA synthesis in HT-1080 or HEK293T, indicating that rDNA transcription requires PinX1 (Fig. S2, *B*–*E*). Pol I transcription activity was directly assessed by an *in situ* assay based on the incorporation of 5-ethynyl uridine (EU). As shown in Figure 2, *F* and *G*, both knockdown and knockout of PinX1 led to the reduction of foci in nucleoli as demonstrated by 5-EU-labeled nascent RNAs. Moreover, we visualized cell protein synthesis through the incorporation of puromycin into nascent proteins. PinX1 depletion significantly led to a decrease in protein synthesis rate (Fig. 2*H*). In collection, these data demonstrated that PinX1 deficiency impairs nucleolar transcription, leading to compromised ribosome biogenesis.

### Nutrient starvation-induced acetylation of PinX1 inhibits rDNA transcription

**Figure 3:**
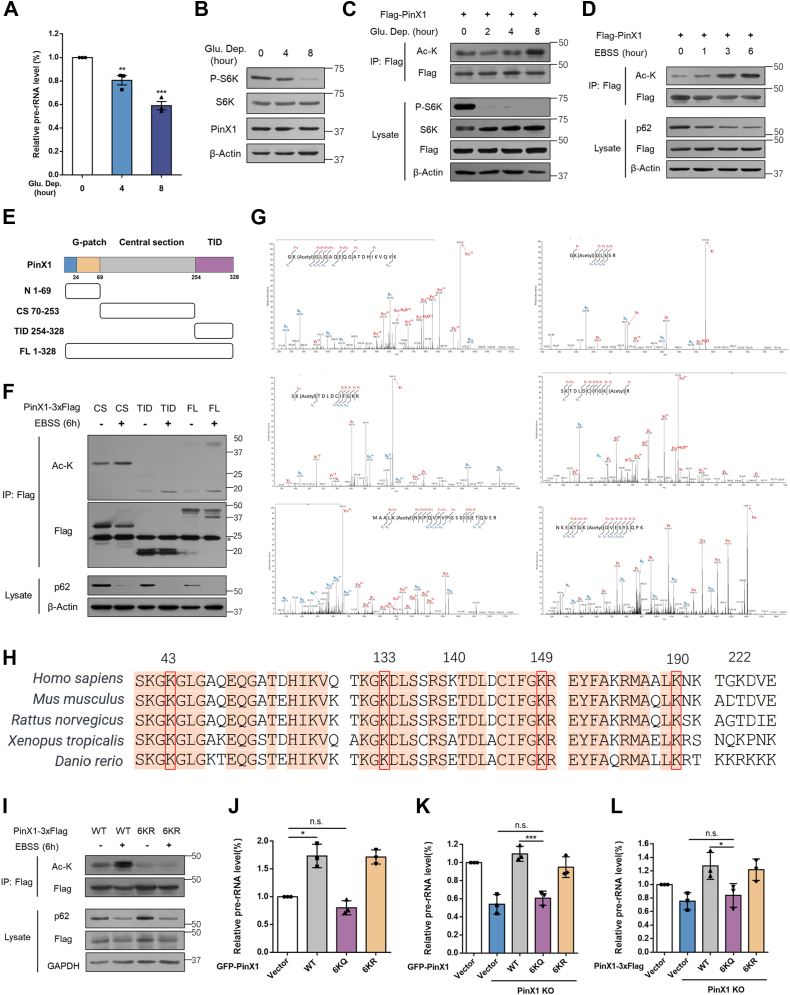
**Nutrient starvation-induced acetylation of PinX1 inhibits rDNA transcription**. *A*, HCT116 cells were cultured in glucose-free medium for the indicated amounts of time (hours). The cells were harvested and pre-rRNA levels were determined by RT-qPCR. Data were analyzed by one-way ANOVA. Error bars indicate the mean ± SD (n = 3). ∗∗*p* < 0.01, ∗∗∗*p* < 0.001. *B*, HCT116 cells were harvested and total proteins were subjected to Western blot for evaluating the indicated proteins. *C* and *D*, HCT116 cells were transfected with Flag-PinX1 plasmid and treated with the indicated time of glucose free medium (*left*) or EBSS medium (*right*). Whole cell lysates were prepared for immunoprecipitation and acetylation of PinX1 was detected by pan-acetyl-lysine antibody. *E*, the schematic diagram represents the PinX1 deletion mutant constructs. *F*, HCT116 cells were transfected with 3×Flag C-terminal-tagged PinX1 deletion mutant plasmids and treated with the indicated time of EBSS medium. The *asterisk* points to the light chain of igG. *G*, anti-Flag immunoprecipitations were performed with the whole cell lysates derived from four 10 cm dishes of HCT116 cells with or without EBSS medium for 6 h. After SDS-PAGE gel separating and Coomassie blue staining, the specific band for PinX1-3×Flag was analyzed by mass spectrometry. The MS/MS spectrums of modified K133, K140, K149, K190, K222 are shown respectively. *H*, the sequences of PinX1 in different species were aligned. The evolutionarily conserved sequences and lysine residues were highlighted. *I*, HCT116 cells were transfected with PinX1-3×Flag WT or 6KR mutant plasmids and treated with the indicated time of EBSS medium. *J*, pre-rRNA levels were determined by RT-qPCR in HT-1080 cells transiently expressing GFP-PinX1 WT, 6KR or 6KQ. (n = 3). *K*, pre-rRNA levels were determined by RT-qPCR in PinX1 WT, PinX1 KO, PinX1-KO HCT116 cells transiently expressing GFP tagged PinX1 WT, 6KR or 6KQ for 48h. (n = 3). *L*, pre-rRNA levels were determined by RT-qPCR in PinX1 WT, PinX1 KO, PinX1-KO HCT116 cells stably expressing PinX1 WT, 6KR or 6KQ. qPCR data were analyzed using one-way ANOVA. Error bars indicate the mean ± SD (n = 3). ∗*p* < 0.05, ∗∗∗*p* < 0.001, n.s. not significant.

As expected, rDNA transcription can be fine-tuned in response to glucose deprivation (Fig. 3*A*), whereas the expression of PinX1 showed no corresponding reduction (Fig. 3*B*). We noticed that acetylation of PinX1 was among the top hits in our previous cellular starvation acetylome screen (6). To confirm it can be regulated by acetylation in response to nutrient starvation, we expressed Flag-tagged PinX1 in HCT116 cells then treated cells with glucose-free medium or Earle’s Balanced Salt Solution (EBSS, deprivation of amino acids). Acetylation of PinX1 was increased in a time-dependent manner (Fig. 3, *C* and *D*). In addition, we found that acetylation level of endogenous PinX1 was elevated in EBSS treated cells (Fig. S3*A*). To examine whether PinX1 acetylation was regulated under other cellular stresses, cells were treated with different agents. However, endoplasmic reticulum stress, serum starvation or DNA damage stress treatment had no significant effect on PinX1 acetylation (Fig. S3, *B*–*D*), demonstrating that PinX1 acetylation only responds to specific nutrients.

To identify potential acetylation sites on PinX1, we constructed PinX1 truncated fragments according to its distinctive domains reported (Fig. 3*E*) (39). We expressed 3×Flag-tagged truncations in HCT116 cells and treated cells with EBSS. All the truncations showed the elevated acetylation signals under the EBSS treatment (Fig. 3*F*), except for the N-terminal truncation which was unstable to express (40). We treated cells with EBSS and purified the acetylated 3×Flag tagged PinX1 protein from HCT116 cells and determined its acetylation sites *via* mass spectrometry. Results showed that lysine (K) residues 43, 133, 140, 149, 190 and 222 were the major acetylation sites (Fig. 3*G*). Conservation analysis of PinX1 indicated that most of the acetylated lysine residues are evolutionarily conserved sites among species (Fig. 3*H*). To validate these lysine residues, we replaced lysine 43, 133, 140, 149, 190, 222 by arginine (6KR) to mimic hypoacetylation at one plasmid and expressed it in HCT116. EBSS treatment could increase acetylation levels of wild-type PinX1, but not 6KR mutant (Fig. 3*I*), further confirming that nutrient starvation induces acetylation of PinX1 at K43, K133, K140, K149, K190 and K222 residues.

To assess the functional consequence of PinX1 acetylation in pre-rRNA synthesis, we ectopically expressed PinX1 wild-type, 6KR or 6KQ (lysine to glutamine mutant for protein hyperacetylation mimic) in HT-1080 and HEK293T cells. PinX1 wild-type or 6KR activated pre-rRNA synthesis while 6KQ did not (Figs. 3*J* and S3, *E*–*G*). To confirm this observation, we transiently re-expressed PinX1 WT, 6KR, or 6KQ in PinX1 knockout HCT116 cells, the results showed that both WT and 6KR could rescue pre-rRNA synthesis of PinX1-KO cells, but not 6KQ (Figs. 3*K* and S3*H*). To further confirm these results, we constructed PinX1-KO HCT116 cells stably expressing PinX1 WT, 6KR, or 6KQ and found that both WT and 6KR could rescue pre-rRNA synthesis of PinX1-KO cells, but not 6KQ (Figs. 3*L* and S3*I*). Taken together, these results demonstrated nutrient starvation-induced acetylation of PinX1 at lysine 43, 133, 140, 149, 190, and 222 inhibits its role in pre-rRNA synthesis.

### CBP/p300 acetylate PinX1 at lysine 43, 133, 140, 149, 190, and 222

**Figure 4:**
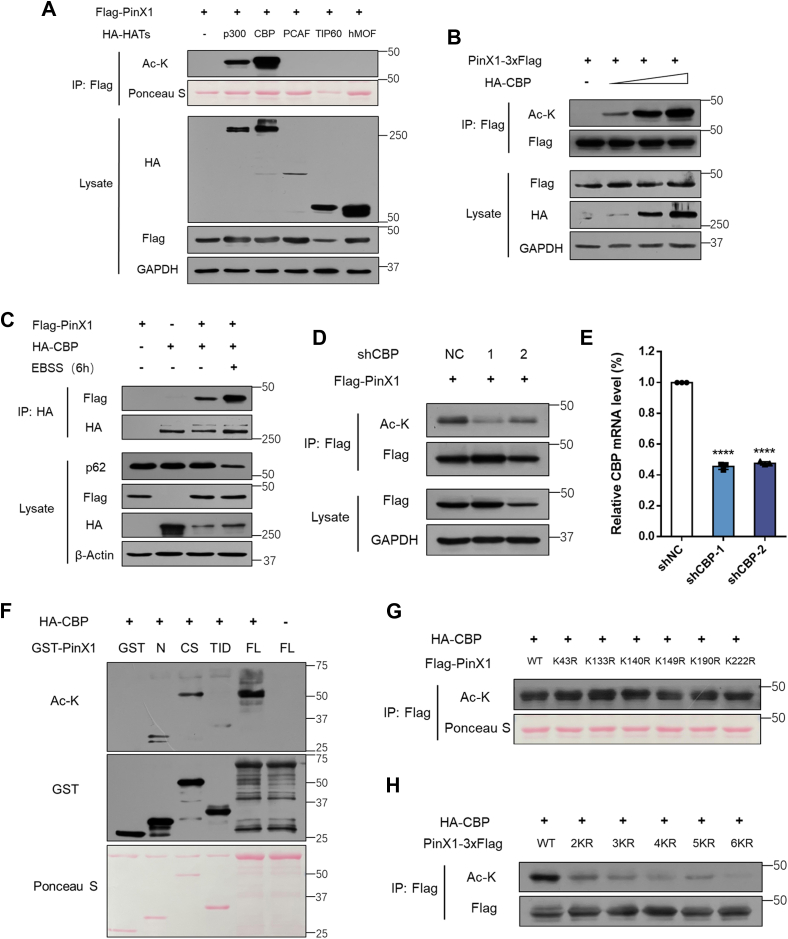
**CBP/p300 acetylate PinX1 upon nutrient starvation**. *A*, HEK293T cells were transfected with plasmids as indicated, and the acetylation of PinX1 was detected. *B*, HEK293T cells were transfected PinX1-3×Flag plasmids together with different amount of HA-CBP, and acetylation of PinX1 was detected. *C*, HCT116 cells were transfected with plasmids as indicated with or without EBSS treatment, and the protein was extracted for co-IP to determine the interaction between PinX1 and CBP. *D* and *E*, HCT116 cells were infected by shCBP lentivirus for 48 h and mixture cells were selected by 1 mg/ml puromycin for 1 week. Acetylation levels of PinX1 were detected and total RNA was subjected to RT-qPCR for evaluation of the mRNA levels of CBP. Error bars indicate the mean ± SD (n = 3). ∗∗∗∗*p* < 0.0001. *F*, GST, GST-PinX1 full-length (FL), and three GST-PinX1 deletion fragments were purified from bacteria transformed with plasmids as indicated. HA-CBP was purified from HEK293T cells transfected with HA-tagged CBP plasmids for 48 h. *In vitro* acetylation assay was performed. GST protein was used as a negative control. *G* and *H*, HEK293T cells were transfected with plasmids as indicated for 24 h. Whole-cell lysates were immunoprecipitated with anti-Flag antibody resin and acetylation of PinX1 was detected. 2KR (K43R, K133R), 3KR (K43R, K133R, K190R), 4KR (K43R, K133R, K190R, K222R), 5KR (K43R, K133R, K140R, K190R, K222R), 6KR (K43R, K133R, K140R, K149R, K190R, K222R). HA, hemagglutinin; HAT, histone acetyltransferase.

To identify the histone acetyltransferases (HATs) that acetylates PinX1, we co-expressed Flag-PinX1 with HA-p300, HA-CBP, HA-PCAF, HA-TIP60 or HA-hMOF in HEK293T cells and examined the acetylation state of Flag-PinX1. We found that the major acetyltransferases for PinX1 are CBP and p300 (Fig. 4*A*). Since CBP gave the strongest signal for PinX1 acetylation, we used CBP for the ensuing test. CBP acetylated PinX1 in a dose-dependent manner (Fig. 4*B*). EBSS treatment increased the interaction of CBP with PinX1 (Fig. 4*C*). To validate CBP-dependent acetylation of PinX1, we knocked down endogenous CBP with shRNA. Down regulation of CBP led to hypoacetylation of PinX1, supporting the notion that CBP acetylates PinX1 (Fig. 4, *D* and *E*). Besides, *in vitro* acetylation assay revealed significant acetylation signal of central section and full-length of PinX1 (Fig. 4*F*), consistent with the mass spectrometry results. To validate CBP-dependent acetylation of PinX1 at these lysine residues, we replaced lysine 43, 133, 140, 149, 190, 222 by arginine (K43R, K133R, K140R, K149R, K190R, and K222R), respectively, and examined acetylation of wild-type and mutant PinX1 after co-expression with HA-CBP. There was no difference in acetylation levels among these mimic mutants respectively (Fig. 4*G*). However, 6KR mutant showed significantly decreased acetylation level compared to wild-type PinX1 (Fig. 4*H*), suggesting that all six sites contribute to PinX1 acetylation by CBP. Collectively, these results demonstrated that acetylation of PinX1 increased in response to nutrient starvation is CBP dependent.

### HDAC3 and HDAC10 deacetylate PinX1

**Figure 5:**
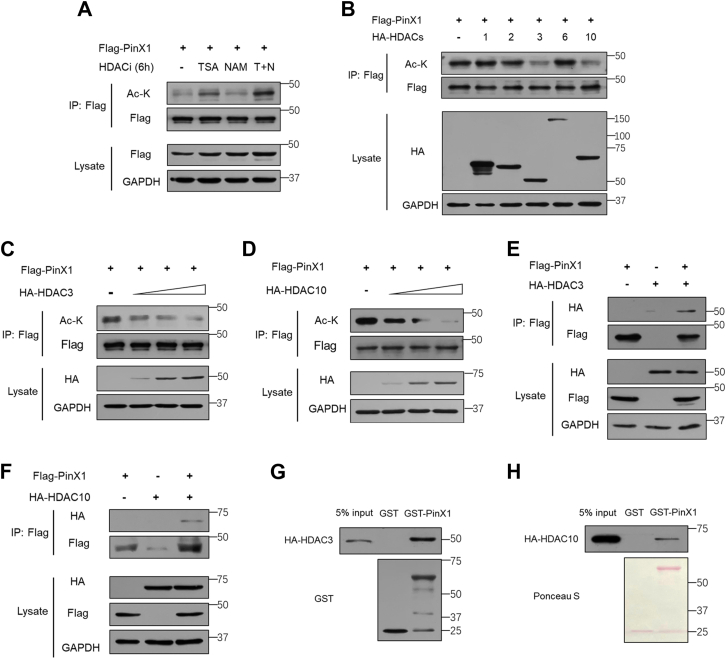
**HDAC3 and HDAC10 deacetylate PinX1**. *A*, HCT116 cells transfected with Flag-PinX1 were treated with TSA (1 mM) or NAM (10 mM) for 6 h before harvest. *B*, HEK293T cells were transfected with plasmids as indicated. PinX1 protein was purified and immunoblotted by pan-acetyl-lysine antibody. *C* and *D*, Flag-tagged PinX1 was co-transfected with different amounts of HA-tagged HDAC3 or HDAC10 in HEK293T cells. *E* and *F*, HEK293T cells were transfected with plasmids as indicated, and the protein was extracted for co-IP to detect the interaction between PinX1 and HDAC3 or HDAC10. *G* and *H*, GST pull-down assay was performed as indicated. GST-PinX1 bound HA-HDAC3 or HA-HDAC10 was evaluated by Western blot using an anti-HA antibody.

Since lysine acetylation is a reversible and dynamic process, it promoted us to identify the deacetylase of PinX1. We evaluated the acetylation levels of PinX1 upon the treatment of class I and II histone deacetylases (HDACs) inhibitor, trichostatin-A (TSA), or an inhibitor of class III deacetylases, nicotinamide (NAM). TSA, but not NAM, significantly increased acetylation of PinX1 (Fig. 5*A*), indicating that HDACs, rather than Sirtuins, are the major deacetylases of PinX1 in cells. We then co-expressed PinX1 with HDACs mainly located in nucleus, including HDAC1, 2, 3, 6, and 10, and examined the acetylation levels of PinX1. We found that overexpression of both HDAC3 and HDAC10 resulted in reduction of PinX1 acetylation (Fig. 5*B*). Both HDAC3 and HDAC10 markedly decreased the acetylation of PinX1 in a dose-dependent manner (Fig. 5, *C* and *D*). Moreover, the interaction of PinX1 with HDAC3/HDAC10 was confirmed by coimmunoprecipitation assay (Fig. 5, *E* and *F*). The *in vitro* GST pull-down assay confirmed that the interaction between PinX1 and HDAC3/HDAC10 is direct (Fig. 5, *G* and *H*). Collectively, these results demonstrated that PinX1 is a substrate of HDAC3 and HDAC10 for deacetylation.

### PinX1 interacts with POLR1G to activate the assembly of Pol I preinitiation complex

To decipher the mechanism by which acetylation of PinX1 impairs pre-rRNA synthesis, we hypothesized that acetylation could alter the localization of PinX1. To test this, we performed nuclear and cytoplasmic fraction separation experiments and found that wild-type, 6KR or 6KQ mutant still localized in nucleus (Fig. S4*A*). Additionally, neither nutrient starvation nor CBP overexpression had effects on nuclear and cytoplasmic fraction of PinX1 (Fig. S4*B*).

**Figure 6:**
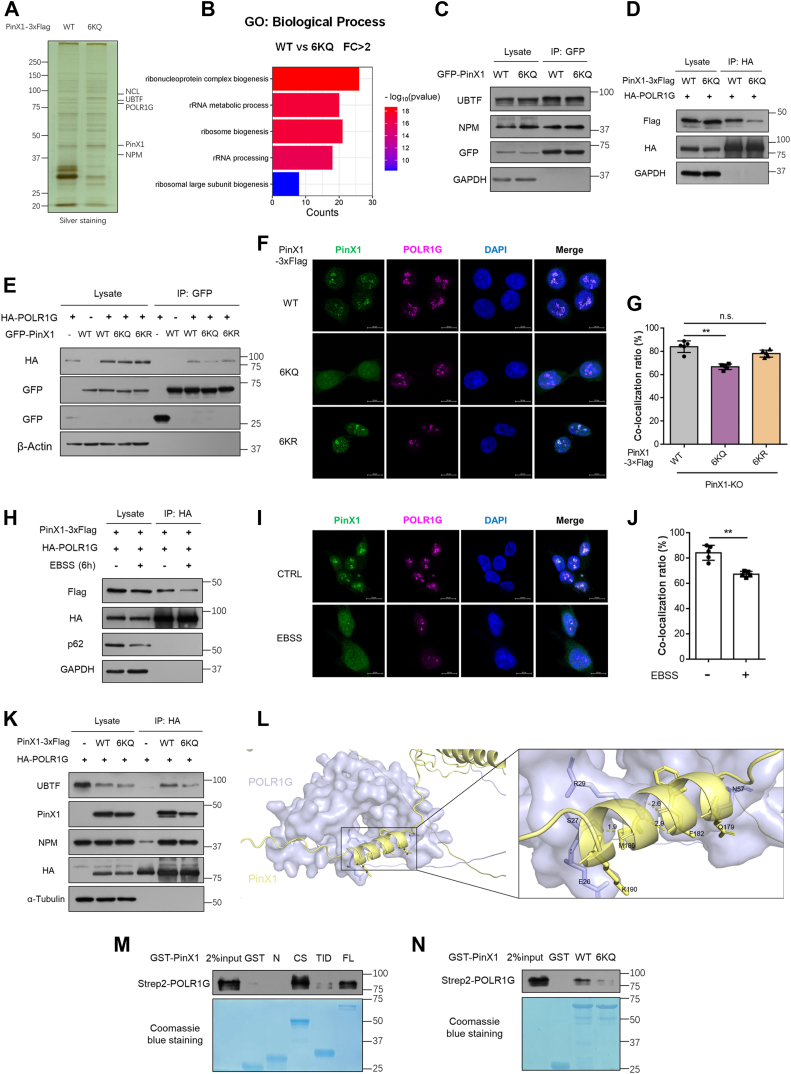
**PinX1 interacts with POLR1G and is required for the assembly of Pol I preinitiation complex**. *A*, HCT116 cells were transfected with 3 × Flag tagged PinX1 WT or 6KQ mutant plasmids. After 48 h, the cells were harvested, and whole cell extracts were immunoprecipitated with an anti-Flag antibody affinity resin. PinX1 and its interacting proteins were eluted by SDS-PAGE, detected by silver and analyzed by mass spectrometry. *B*, the bar graph shows the enrichment analysis of PinX1-assocaited proteins in the terms of GO biological process. FC, fold change. *C*, HEK293T cells were transfected with GFP tagged PinX1 WT or 6KQ plasmids, and the proteins were extracted for co-IP to detect the interaction with endogenous UBTF and NPM, which were from mass spectrometry results. *D* and *E*, HEK293T cells were transfected as indicated, and the protein was extracted for co-IP to detect the interaction of PinX1 and POLR1G. *F*, PinX1 WT, 6KR, 6KQ rescued HCT116 cells were stained with PinX1 antibody (*green*) and POLR1G antibody (*magenta*). DAPI (*blue*), nucleus. Scale bar = 10 μm. *G*, the quantification of co-localization ratio of PinX1 and POLR1G, related to (*F*). The data was represented by mean ± SD (n = 5). ∗∗*p* < 0.01. *H*, HCT116 cells were transfected with PinX1-3 × Flag and HA-POLR1G plasmid and treated with the indicated time of EBSS medium. The protein was extracted for co-IP to detect the interaction between PinX1 and POLR1G. *I*, HCT116 cells were treated with or without EBSS medium and stained with PinX1 antibody (*green*) and POLR1G antibody (*magenta*). DAPI (*blue*), nucleus. Scale bar = 10 μm. *J*, the quantification of co-localization ratio of PinX1 and POLR1G, related to (*I*). The data was represented by mean ± SD (n = 5). ∗∗*p* < 0.01. *K*, HA-POLR1G was transfected into PinX1 knockout HCT116 cells individually or together with PinX1 WT or 6KQ. The protein was extracted for co-IP to detect the interaction between POLR1G and other components among PIC. *L*, the predicted structure of human PinX1 and POLR1G from the AlphaFold2 database (identifier: AF-Q96BK5-F1, AF-O15446-F1) and docking model was visualized in PyMOL. *M*, GST pull-down assay was performed as indicated. GST-PinX1 truncation bound Strep2-POLR1G was evaluated by Western blot using an anti-POLR1G antibody. *N*, GST-PinX1 6KQ bound Strep2-POLR1G was evaluated.

We then investigated if PinX1 acetylation can affect its associated proteins by purifying PinX1 wild-type and 6KQ mutant interacting proteins. The purified proteins were visualized by silver staining (Fig. 6*A*) and identified by mass spectrometry. PinX1 WT interacts with multiple proteins that function in ribosome biogenesis, which 6KQ captured less (Fig. 6*B*). We reviewed these two interactome results (vector *versus* WT, WT *versus* 6KQ) and focused on the PinX1 interacting proteins associated with rDNA transcription, rRNA processing or maturation. To confirm the interaction between PinX1 and its counterparts, we performed co-IP tests and found that endogenous UBTF, NPM and NCL could be immunoprecipitated by ectopically expressed PinX1 in HEK293T cells (Figs. 6*C* and S4*C*). Moreover, we transiently transfected HA-POLR1G into PinX1-3×Flag expressed HEK293T cells and found that POLR1G could be clearly co-immunoprecipitated by PinX1 WT, but not 6KQ (Fig. 6*D*). And then we transiently transfected HA-POLR1G or HA-DDX21 into GFP-PinX1 expressed HEK293T cells and found that both POLR1G and DDX21 could be clearly co-immunoprecipitated (Figs. 6*E* and S4*D*). Intriguingly, only PinX1 6KQ reduced its association with POLR1G. In addition, the immunofluorescent staining assay demonstrated that stable cell lines rescued by PinX1 6KQ significantly decreased the levels of co-localization of PinX1 and POLR1G (Fig. 6, *F* and *G*), suggesting that acetylation of PinX1 may impairs the interaction with POLR1G. In support of this view, we co-expressed PinX1-3×Flag and HA-POLR1G in HCT116 cells and then treated the cells with EBSS which was identified to elevate acetylation levels of PinX1. The interaction between PinX1 and POLR1G was impaired under EBSS treatment (Fig. 6*H*). Furthermore, much less POLR1G was co-immunoprecipitated by acetylated PinX1 which was induced by CBP overexpression (Fig. S6*A*). As shown in Figure 6, *I* and *J*, EBSS treatment decreased the co-localization of endogenous PinX1 and POLR1G. These results indicated that nutrient starvation-induced acetylation of PinX1 impairs its interaction with POLR1G. Given that 6KQ does not rescue pre-rRNA synthesis of PinX1-KO cells, we examined the interaction of UBTF with other components of Pol I PIC in PinX1 rescued cells. We found that exogenous POLR1G failed to coimmunoprecipitate endogenous UBTF in PinX1-KO cells. Re-expressed PinX1 WT recovered the interaction of POLR1G with UBTF, while 6KQ partially recovered (Fig. 6*K*), indicating that as an adapter, hyperacetylated PinX1 reduced the recruitment of POLR1G to UBTF for the assembly of Pol I PIC.

In order to gain the mechanism why acetylation of PinX1 weakened the interaction with POLR1G, we sought to determine the structural base of interaction between PinX1 and POLR1G. Due to the lack of the intact crystal structure of POLR1G or PinX1 for homology modeling, we obtained the predicted structure of PinX1 (Identifier: AF- AF-Q96BK5-F1) and POLR1G (AF-O15446-F1) from AlphaFold2 and performed protein-protein docking simulation. The lowest-energy binding conformation of PinX1 with POLR1G demonstrated that, PinX1 bound to the pocket of POLR1G through hydrogen bonds *via* its Q179, F182, M186, and salt bridge *via* its K190 (Fig. 6*L*). We also obtained the predicted interacting interface of PinX1-C terminal with POLR1G, which displays low affinity though (Fig. S6, *B* and *C*). To verify this finding, GST pull-down experiments were performed with purified GST-PinX1 including its truncations or 6KQ mutation constructs as well as Strep2-tagged POLR1G. We found that full length GST-PinX1 bound to Strep2-POLR1G *in vitro* (Fig. 6*M*). More specifically, PinX1-binding regions of POLR1G were narrowed down to the central section (aa 70–253), which contains most acetylated lysine residues. However, GST-PinX1 6KQ reduced its direct binding to POLR1G (Fig. 6*N*), suggesting that loss of capability to bind POLR1G results from the alteration of charge. Given that depletion of POLR1G can also result in the inhibition of rDNA transcription, we checked the expression levels of POLR1G in PinX1 rescued cells. The expression levels of POLR1G had no difference among PinX1 knockout and rescued cells (Fig. S5*C*), suggesting that interaction between PinX1 and POLR1G is a response to the change in rRNA synthesis. Collectively, these results reveal that acetylation of PinX1 regulates the assembly of Pol I PIC by maintaining the interaction of POLR1G with UBTF.

### Hyperacetylated PinX1 represses protein synthesis and cell proliferation

**Figure 7:**
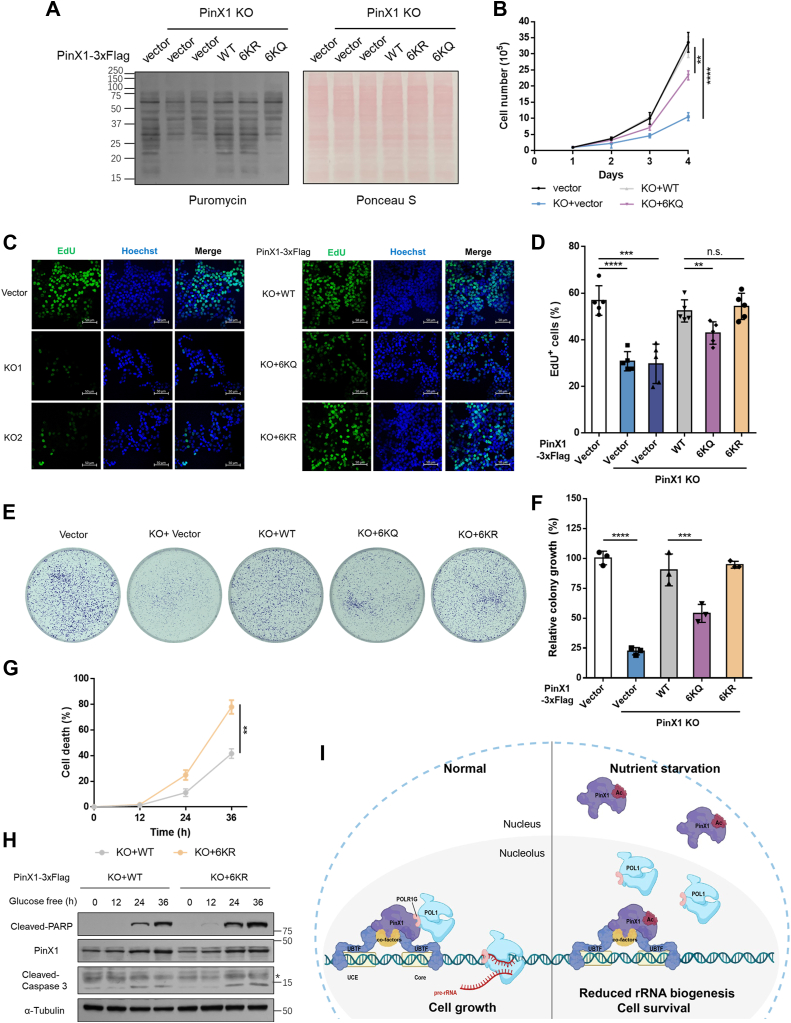
**Hyperacetylated PinX1 represses protein synthesis and cell proliferation.***A*, PinX1 wild type, PinX1 KO and rescued HCT116 cells were pulse treated with puromycin (10 mg/ml, 30 min) before harvest. Lysates were analyzed by Western blotting. Ponceau S staining was used as the loading control. *B*, wild-type, PinX1 KO or PinX1 rescued HCT116 cells were seeded into 6-well plates at 10^5^ cells per well and the cell numbers were counted every day. *C*, the representative image of EdU incorporation assay in wild type, PinX1 KO and rescued HCT116 cells. Scale bars = 50 μm. *D*, the percentage of cells positively stained with EdU was quantified. Five fields of cells for each sample were counted and analyzed using one-way ANOVA. Data are shown as mean ± SD (n = 5). ∗∗*p* < 0.01, ∗∗∗∗*p* < 0.0001, n.s. not significant. *E*, wild-type, PinX1 knockout, or rescued HCT116 cells as indicated were seeded into 6 cm dishes at 5000 per dish, and the cell colonies were stained with crystal violet after 7 days. *F*, colony numbers were quantitated. Data are shown as mean ± SD of triplicate experiments. ∗∗∗*p* < 0.001, ∗∗∗∗*p* < 0.0001. *G*, PinX1 WT or 6KR rescued HCT116 cells were cultured in glucose free medium for the indicated time (hours), followed by cell viability analyses using PI uptake assay. The percentage of cells positively stained with PI was quantified using unpaired *t* test. Data are shown as mean ± SD (n = 3), with ∗∗*p* < 0.01. *H*, the levels of proteins were determined by Western blotting as indicated. The asterisk points to a nonspecific band. *I*, working model illustrates that acetylation of PinX1 regulates rDNA transcription by impairing the interaction of POLR1G with UBTF.

PinX1 depletion significantly led to a decrease in protein synthesis rate, which was rescued in PinX1 WT or 6kR cells (Fig. 7*A*), indicating that acetylation of PinX1 suppresses ribosome biogenesis. Consistently, we found that depletion of PinX1 decreased cell proliferation rate of HCT116 cells, which was completely rescued by WT PinX1, but partially by 6KQ (Fig. 7*B*). Besides, depletion of PinX1 resulted in fewer EdU labeling cells, which was completely rescued by WT or 6KR PinX1, but partially by 6KQ (Fig. 7, *C* and *D*), indicating that repressed protein synthesis is corelated with impaired proliferative capacity. Additionally, 6KQ partially rescued the ability of colony formation of PinX1-KO HCT116 cells (Fig. 7, *E* and *F*). These results demonstrated that the acetylation of PinX1 is negatively associated with cell proliferation *via* suppressing ribosome biogenesis.

To determine the role of the acetylation of PinX1 regarding the stress condition, cells were incubated with glucose free medium (unlike EBSS, cells can adapt and survive up to 36 h in glucose free medium) for various lengths of time and stained with propidium iodide (PI). Compared with PinX1 wild type cells, PinX1 6kR cells were more sensitive to glucose deprivation-induced cell death (Figs. 7*G* and S7*A*), as marked by the cleavage of PARP and caspase-3 (Fig. 7*H*). Taken together, as a strategy to save energy, glucose deprivation-induced acetylation of PinX1 facilitate cell survival.

## Discussion

Regulation of ribosome biogenesis is one of the strategies for cells surviving in response to nutrient starvation. However, the mechanism orchestrating ribosome biogenesis with cellular nutrition status remains unclear. In this study, we found PinX1 and its acetylation play very important roles in regulating ribosome biogenesis. Depletion of PinX1 impairs rDNA transcription, compromises ribosome biogenesis and inhibits tumor cells proliferation. In normal condition, PinX1 associates with UBTF and directly binds to POLR1G which is required for the assembly of Pol I PIC. Upon nutrient starvation, PinX1 is acetylated, which hinders its binding to POLR1G leading to disassembly of PIC and reduced rRNA biogenesis (Fig. 7*I*). Thus, our findings uncover a novel role of PinX1 and its acetylation, fine-tuning nucleolar transcription to stress signaling.

Post-translational modifications play a crucial role in regulating Pol I. Acetylation of POLR1E at lysine 373 by CBP and deacetylation by SIRT7 modulate the association of Pol I with DNA. In response to glucose deprivation, SIRT7 is released from nucleolus, leading to hyperacetylation of POLR1E and decreased Pol I transcription (37). The mTOR signaling pathway, as a regulatory hub, coordinates a multitude of signals with gene expression programs (41). In response to a nutrient-sufficient context, mTOR-S6K1 activation leads to phosphorylation of the initiation factors Rrn3 in yeast cells (42) and RRN3 (43) and UBTF (44) in human cells. Similarly, Growth factors induce rRNA synthesis by phosphorylating S633 and S649 of Rrn3 in a Ras-GTP-MAPK-ERK/RSK signaling cascade in mouse cells (45). Our results revealed that PinX1 interacts with Pol I and acetylation of PinX1 orchestrates Pol I transcription with cellular nutrition status. However, the other associated factors of Pol I as well as Pol I itself were not captured in our previous acetylome screen (6), suggesting that as an only component of PIC in response to nutrition starvation with raised acetylation levels, PinX1 is the critical sensor for cellular nutrition status for cell survival.

Nucleolus acts as a cellular stress receptor. Nucleolar stress occurs in various stressful events, such as ionizing irradiation, hypoxia, heat shock, nutrient starvation and DNA damage, and consequently downregulates rDNA transcription. These stressful cues lead to a series of events, such as morphological as well as componential changes in nucleolus, translocation of nucleolar proteins into the nucleoplasm and p53 accumulation (46). To test whether PinX1 acetylation accounts for the reduction of rDNA transcription in these stressful events, we treated cells with endoplasmic reticulum stress or replication stress agents (Fig. S3, *B* and *D*). The results showed that these agents had no effect on PinX1 acetylation, indicating that other type stress events-induced downregulation of rDNA transcription may not be regulated by PinX1 acetylation. Results showed that targeting CBP/p300 by using a CBP/p300 inhibitor, A-485 sensitized HCT116 to glucose deprivation (Fig. S7, *B*–*D*), indicating that nucleolus developed a CBP/p300-dependent pathway in response to the specific stress.

Because TRF1 and hTERT are two classical targets of PinX1, we also checked their interaction with PinX1. TRF1, as a component of the Shelterin complex, is required for telomere protection and chromosome stability. It has been reported that PinX1 stabilizes TRF1 in HeLa cells and PinX1 depletion inducing DNA damage responses at telomeres was mediated by TRF1 degradation (47). PinX1 depletion HCT116 cells showed multiple obvious γH2AX foci by immunofluorescent staining (Fig. S5*A*) and accumulation of γH2AX as well p21 by Western blotting (Fig. S5*B*) as expected. We found that neither depletion nor acetylation of PinX1 activate the degradation of endogenous or exogenous TRF1 in HCT116 cells (Fig. S5, *C* and *D*), indicating that PinX1 regulating the stabilization of TRF1 is in a context-dependent manner. Besides, we compared the interaction of hTERT with wild-type PinX1 or 6KQ mutant. Both wild-type and 6KQ mutant were associated with hTERT at similar levels (Fig. S5*E*), demonstrating that acetylation does not affect the interaction of PinX1 with hTERT.

It has been reported that half of PinX1 was detected in the nucleolus and the other in nucleoplasm in many cell types. In addition, Yoo *et al.* found that residues 150 to 328 are required for nucleolar localization of hPinX1 (9). We found that PinX1 6KQ significantly increased its localization to the nucleoplasm in PinX1 re-expressed stable cells (Fig. 6*F*). Moreover, endogenous PinX1 was distributed to the nucleoplasm under EBSS treatment (Fig. 6*I*), which was consistent with the 6KQ construct. These results suggest that acetylation may impact on the shuttles of PinX1 between nucleolus and nucleoplasm.

Given that PinX1 is localized to nucleolus and interacts with POLR1G, two possibilities emerged for the mechanism by which the interaction of PinX1 with POLR1G altered. Either hyperacetylated PinX1 translocated to nucleoplasm and led to its inaccessible to POLR1G, or hyperacetylated PinX1 inhibited its binding to POLR1G directly. To differentiate between these possibilities, we performed GST pull-down experiments and found that PinX1 6KQ binds POLR1G much weaker compared with wild-type (Fig. 6*N*), indicating that acetylation of PinX1 lysine residues altered charge status and dampened its direct binding to POLR1G. Since endogenous POLR1G was localized to nucleolus stably, it is safe to claim that acetylation of PinX1 leads to both inaccessible and weaker binding with POLR1G. Though K190 of PinX1 is acetylated and responsible for the interaction between PinX1 and POLR1G, the interaction between PinX1 and POLR1G does not change when mutating K190 to Q or R merely (Fig. S6*D*), indicating that all the six acetylated lysine residues contribute to affect the molecular interaction in an undefined manner. Future work will be focused on the molecular structure and potential inhibitors which can repress ribosome biogenesis by interfering with the interaction between PinX1 and POLR1G.

## Experimental procedures

### Cell lines

HEK293T, HT-1080 and colon cancer cell lines in this study were obtained from American Type Culture Collection (ATCC). HCT116, HEK293T and HT-1080 cells were cultured in DMEM (Thermo Fisher Scientific) supplemented with 10% fetal bovine serum and 1% penicillin/streptomycin (Thermo Fisher Scientific). All the cells were cultured at 37 °C in a humidified 5% CO_2_ incubator.

### Protein extraction and western blotting

For protein extraction, harvested cells were lysed in Flag lysis buffer [50 mM Tris–HCl (pH 8.0), 137 mM NaCl, 1% Triton X-100, 0.2% Sarkosyl, 1 mM NaF, 1 mM Na3VO4, and 10% glycerol] containing protease inhibitor cocktail, 1 mM phenylmethyl sulfonyl fluoride and 1 mM dithiothreitol for 60 min on ice. The supernatant was collected after centrifugation at 15,000 rpm for 15 min. Protein concentrations were determined by Bradford protein assay (Biorad). Equal concentrations of protein were diluted into 1×SDS-PAGE loading buffer, boiled at 95 °C for 5 min and loaded on eight to 15% SDS-PAGE gels. Proteins were transferred onto nitrocellulose membranes (GE HealthCare). Next, Ponceau staining was used to check the quality of protein transfer. Membranes were blocked at room temperature for 1 h in 5% skim milk and then incubated at 4 °C overnight with the indicated antibodies diluted in 5% BSA or 5% skim milk. All western blotting solutions were prepared in 1×TBST. Anti-PinX1 (1:10000), anti-p62 (1:10000), anti-GFP-Tag (1:50000), anti-HA-Tag (1:10000), anti-POLR1G (1:10000), anti-Acetylated-Lysine (1:1000), anti-Phospho-mTOR (1:1000), anti-mTOR (1:1000), anti-Phospho-p70 S6 Kinase (1:1000), anti-p70 S6 Kinase (1:1000), anti-GAPDH (1:1000), anti-GST-Tag (1:50000), anti-Cleaved PARP (1:1000), anti-Phospho-Histone H2A.X (1:1000), anti-p21 (1:1000), anti-β-Actin (1:1000), anti-α-Tubulin (1:1000), anti-UBTF (1:1000), anti-NPM (1:1000), anti-NCL (1:1000), anti-GRP78 (1:1000), anti-FLAG (1:50000), anti-Puromycin (1:10000), anti-Histone H3 (1:5000), and anti-TRF1 (1:1000). The membranes were washed four times for 5 min with 1×TBST and then incubated at room temperature for 1 h with the corresponding secondary antibody (Jackson, 1:5000). The membranes were washed four times with 1×TBST and detected with ECL solution (Thermo Fisher Scientific).

For nuclear and cytoplasmic protein extraction, cells were treated as indicated, and proteins were extracted using the Nuclear and Cytoplasmic Protein Extraction Kit (Beyotime) according to the manufacturer’s instructions.

### Immunoprecipitation and co-immunoprecipitation

For immunoprecipitation, cells were lysed in Flag lysis buffer. The supernatants were incubated with anti-Flag M2 agarose beads or anti-HA magnetic beads overnight at 4 °C. After being washed with lysis buffer five times, samples were diluted with 1× loading buffer and proteins were eluted either *via* boiling at 95 °C for 5 min, or corresponding peptides. For elution of the Strep2 tag agarose beads, the extra biotin (10 mM) was added to the buffer and the supernatant was collected.

For co-immunoprecipitation, cells were lysed in BC100 buffer [100 mM NaCl, 20 mM Tris-HCl (pH 7.9), 20% glycerol, and 0.2% NP-40] supplied with protease inhibitor cocktail, 1 mM phenylmethyl sulfonyl fluoride and 1 mM dithiothreitol. The whole cell lysates were incubated with anti-GFP, anti-HA agarose beads at 4 °C overnight, or 2 μg of anti-POLR1G antibody at 4 °C overnight and further with Protein A/G agarose beads for another 2 h. After being washed with lysis buffer five times, the proteins were eluted *via* boiling with 1×loading buffer and then analyzed by western blotting.

### GST pull-down assay

GST-PinX1 proteins were expressed in TSsetta (DE3) bacteria and induced with IPTG, then purified with Glutathione-Sepharose 4B beads (GE Healthcare). Strep2-POLR1G proteins were purified from TSsetta (DE3) bacteria with anti-Strep2 tag protein agarose beads (AlpaLifeBio). HA-HDAC3/HDAC10 were purified from transfected HEK293T cells. GST-PinX1 were incubated with Strep2 or HA proteins in BC200 buffer [200 mM NaCl, 20 mM Tris-HCl (pH 7.9), 20% glycerol, and 0.2% NP-40] at 4 °C overnight. The beads were washed with BC500 (same as BC200, except 500 mM NaCl), BC200 or BC100 buffer and boiled in 1×SDS loading buffer. Proteins were analyzed with anti-GST, anti-POLR1G or anti-HA antibody by Western blotting and Coomassie blue staining.

### Plasmids

PinX1 was cloned into pCMV-Myc, pcDNA3.1 to 3×Flag-C, pEGFP-C2, pGEX-4T-3 and PiggyBac-CMV-MCS-Hgr vectors respectively. POLR1G was cloned into pET45a-Strep2 vector. pML-EnCMV-3×HA-POLR1G-SV40-Neo was obtained from MiaoLingBio, China. CBP, p300, TIP60, PCAF, hMOF, HDAC1, HDAC2, HDAC3, HDAC6 and HDAC10 cloned into pcDNA3.1 were from this lab. PinX1 mutation constructs were generated with a KOD Plus Mutagenesis Kit (TOYOBO).

### shRNA, siRNA, CRISPR-Cas9, and rescued cell lines

To generate CBP knockdown cell lines, short hairpin RNA (shRNA) sequences were ligated into pLKO.1-puro plasmid and co-transfected with viral packaging plasmids psPAX2 and pMD2.G into HEK293T cells. 48 h after transfection, the culture medium was collected and filtered through 0.22 μm strainers. HCT116 cells were infected with the filtered viral supernatant for 48 h and then selected with 1 μg/ml puromycin for 1 week.

siRNA oligonucleotides were transfected into HEK293T and HT-1080 cells using INTERFERin transfection reagent (Polyplus) according to the manufacturer’s instructions. Cells were harvested after 48 h of transfection.

To generate PinX1 knockout cell lines, the sgRNA sequence was ligated into the LentiCRISPRv2 plasmid and then transfected into HCT116 cells. 48 h after transfection, the cells were selected with l μg/ml puromycin for 1 week, and monoclonal cells were seeded into 96-well plates for further cultivation.

For PinX1-rescued cell lines, cDNA of 3×Flag-tagged PinX1 WT, 6KR, and 6KQ mutants were subcloned into PiggyBac-CMV-MCS-Hgr vector. Super PiggyBac Transposase vector was co-transfected with PiggyBac-CMV-MCS-Hgr vector, PiggyBac-PinX1 WT, PiggyBac-PinX1 6KR, or PiggyBac-PinX1 6KQ into PinX1 KO HCT116 cells, respectively. 48 h after transfection, the cells were selected with 50 μg/ml hygromycin for 1 week.

### *In vitro* acetylation assay

GST-PinX1 was purified from bacteria as described in the GST pull-down assay above and eluted with GSH (50 mM). HA-CBP was purified from HEK293T cells *via* anti-HA beads and eluted with HA peptides. Then these two proteins were incubated together in acetylation buffer (20 mM pH 8.0 HEPES, 1 mM dithiothreitol, 1 mM phenylmethyl sulfonyl fluoride, and 0.1 mg/ml BSA) supplemented with 2 mM acetyl-CoA at 37 °C for 30 min. The reaction was stopped by adding loading buffer and boiling at 95 °C for 5 min. Samples were analyzed by Western Blotting.

### Immunofluorescence staining

Cells were seeded on glass coverslips in 6-well plates. After treatment, the cells were fixed with 4% paraformaldehyde (Biosharp) for 20 min and permeabilized with 0.3% Triton X-100 in PBS for 20 min at room temperature. After washing with 0.1% Triton X-100 in PBS for four times, the coverslips were blocked for 1 h with blocking buffer (5% BSA, 3% goat serum, and 0.1% NaN3 in washing buffer) and incubated with indicated antibodies at 4 °C overnight. After rinsing with washing buffer, the cells were incubated with the secondary antibodies conjugated with Alexa-488 or Alexa-594 at room temperature for 1 h, followed by DAPI incubation for 15 min. The cells were observed under a confocal microscope (Zeiss LSM880).

### Mass spectrometry assay

For identification of acetylation sites and PinX1-interacting proteins, 3 × Flag-PinX1 WT or 6KQ was transfected into HCT116 cells. Cells were lysed in BC100 buffer, and PinX1 or PinX1-interacting proteins were enriched using anti-Flag M2 beads. The eluted proteins were resolved on a 10% SDS-PAGE gel and visualized by Coomassie Brilliant Blue staining. Briefly, gel slices were cut into about 1 mm^3^ cubes and destained with 50% (v/v) acetonitrile (ACN) in 50 mM NH_4_HCO_3_. After dehydration by neat ACN, protein disulfide bonds were reduced with 10 mM dithiothreitol (DTT) in 100 mM NH_4_HCO_3_ at 56 °C for 30 min, followed by alkylation with 55 mM iodoacetamide (IAM) in 100 mM NH_4_HCO_3_ at room temperature for 20 min in the dark. In-gel protein digestion was performed with 1.2 ng/μl trypsin and 10% (v/v) ACN in 50 mM NH_4_HCO_3_ for 16 h at 37 °C. The tryptic peptides were extracted from gel cubes twice by incubating with 50% (v/v) ACN and 5% (v/v) formic acid (FA) for 20 min and dried on a SpeedVac vacuum concentrator.

For LC-MS/MS analysis, peptides were separated *via* nanoflow reversed-phase LC on an EASY-nLC 1000 System (Thermo Scientific) using a homemade capillary column (75 μm × 150 mm) packed with 4-μm C18AQ particles (100 Å, Michrom BioResources). The gradient was programmed as follows: solvent B (80% ACN, 0.1% FA) increased linearly from 5% to 35% over 40 min, ramped to 95% in 2 min, and maintained at 95% for 10 min before column re-equilibration with solvent A (97% H_2_O, 3% ACN, 0.1% FA). Eluted peptides were electrosprayed directly into a hybrid linear ion trap-Orbitrap mass spectrometer (LTQ-Orbitrap Velos, Thermo Scientific) operating in data-dependent acquisition mode. Full MS scans (m/z 400–1200) were acquired by the Orbitrap at R = 60,000, followed by CID fragmentation of the top 10 most intense ions (charge state ≥ +2) in the ion trap with 2-Da isolation window and 35% normalized collision energy. Dynamic exclusion was applied with a 30-s repeat duration and 12-s exclusion time.

Raw MS data were processed using Mascot against the *Homo sapiens* proteome (UniProt UP000005640). Database searches specified: trypsin digestion (≤2 missed cleavages), precursor mass tolerance ±20 ppm, fragment mass tolerance ±0.8 Da. Variable modifications included acetylation and oxidation. Carbamidomethylation was set as fixed. Peptide/protein identifications were filtered at FDR < 1% (minimum ratio count = 2). All experiments included ≥3 biological replicates.

### Cell proliferation assay

1 × 10^5^ cells were seeded into 6-well plates. At 1-, 2-, 3-, and 4-day post-seeding, the cells were digested, added into Trypan Blue solution, and counted *via* Countstar IC1000 (Inno-Alliance Biotech).

### Colony formation

1 × 10^5^ cells were seeded into 6-cm dishes and cultured for 1 week. Next, the cells were fixed with 4% paraformaldehyde for 20 min and stained with 0.2% crystal violet solution for 15 min. Stained puncta were counted by Image J software.

### EU and EdU incorporation assay

Cells were seeded on glass coverslips in 6-well plates. After 3 days, all cells were pulsed with 1 mM 5-ethynyl uridine (EU) for 10 min for nascent RNA labeling or 10 μM ethynyl deoxyuridine (EdU) for 2 h for nascent DNA labeling. Then the cells were fixed with 4% paraformaldehyde for 20 min and permeabilized with 0.3% Triton X-100 in PBS for 20 min at room temperature. After washing four times, the cells were subjected to click reaction utilizing a BeyoClick Kit (Beyotime) according to the manufacturer’s protocols.

### RNA isolation and real-time quantitative PCR

Total RNA was extracted with TRIzol reagent (Thermo Fisher Scientific). Next, 1 μg total RNA was transcribed into cDNA utilizing a cDNA Synthesis Kit (Yeasen). The cDNA was diluted for 5-fold and mixed with qPCR SYBR Green Master Mix (Yeasen) containing 0.2 μM primer pairs (10 μM primer pairs stock solution). Real-time quantitative PCR was performed on 7500 Real-time PCR System (Applied Biosystems).

### Statistical analysis

Information regarding the error determination and number of independent biological replicates in experiments (n) can be found for each experiment in the figure legends. Student’s *t* test, Mann-Whitney U test, or one-way ANOVA was used for statistical analyses, and exact *p* values were presented in a separate supplementary file. *p* < 0.05 was considered statistically significant. Not significant (n.s.): *p* > 0.05, ∗: *p* < 0.05, ∗∗: *p* < 0.01, ∗∗∗: *p* < 0.001, ∗∗∗∗*p* < 0.0001. GraphPad Prism was used for statistical analyses.

## Data availability

Any additional information required to reanalyze the data reported in this paper is available from the lead contact upon request.

## Supporting information

Supplementary figures, legends, MS results and reagent details were uploaded as the Supporting information.

## Conflict of interest

The authors declare that they have no conflicts of interest with the contents of this article.
