# Supplementary material for: Acetylation of microtubule-binding PinX1 orchestrates ribosome biogenesis to nutrient starvation via the RNA polymerase I preinitiation complex
Source: J Biol Chem. 2025 Jul 8;301(8):110465. doi: 10.1016/j.jbc.2025.110465 (PMC12329599; doi:10.1016/j.jbc.2025.110465)
Supplement: Supplementary Materials [file mmc1.docx]

Supplementary Materials for

**Acetylation of microtubule-binding PinX1 orchestrates ribosome biogenesis to nutrient starvation via the RNA polymerase I preinitiation complex**


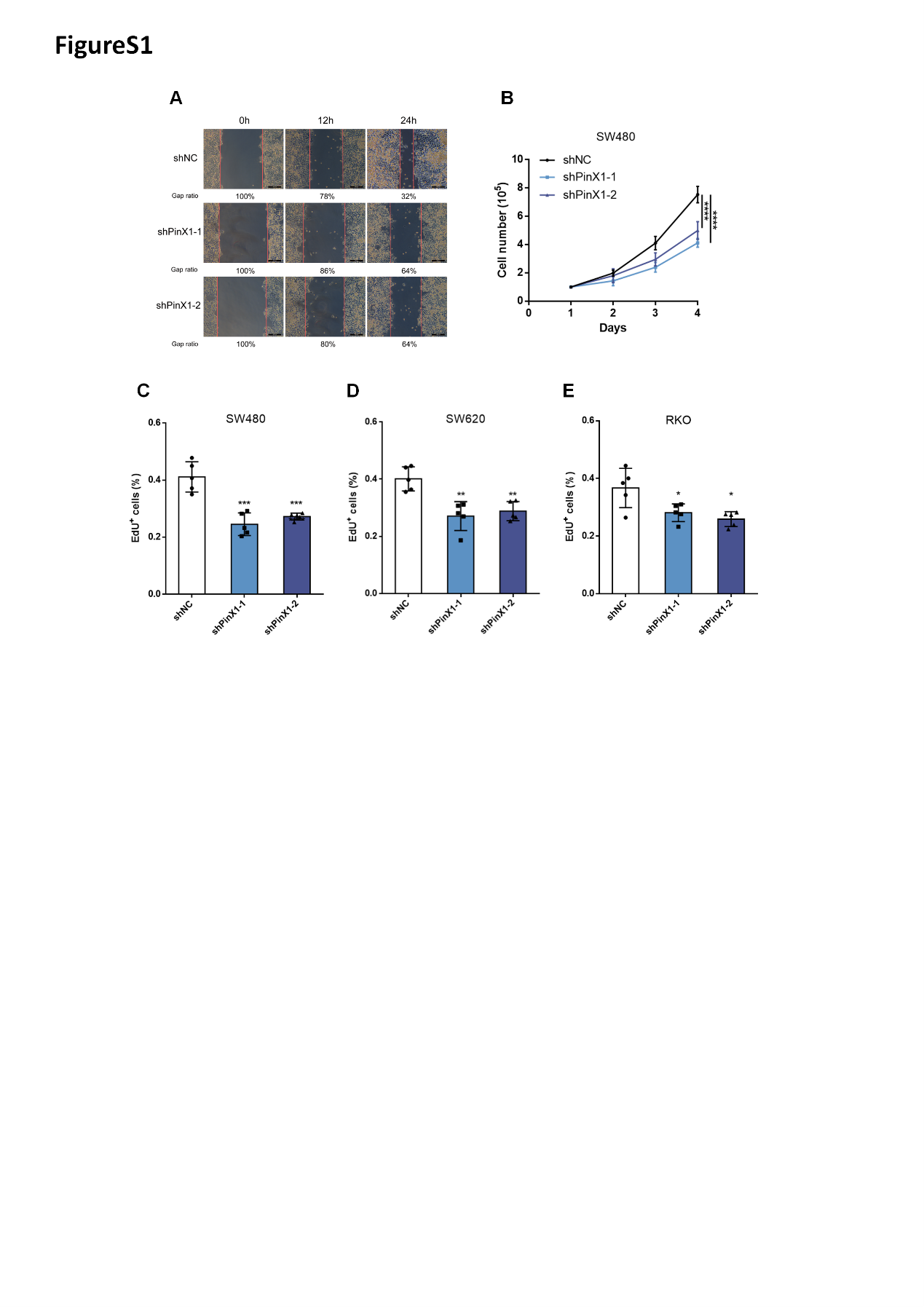


**Fig.s1 PinX1 deficiency inhibits cell migration and proliferation capacity.**

(A) Wound healing assays were performed in SW480 cells (WT, shPinX1-1 or shPinX1-2). Images were captured at times 0, 12, and 24 h. Scale bars = 100 μm. (B) Cell numbers of SW480 cells (WT, shPinX1-1 or shPinX1-2) were counted. (C-E) The percentage of SW480, SW620 or RKO cells positively stained with EdU was quantified. Error bars indicate the mean ± SD (n = 5). **p*< 0.05, ***p*< 0.01, ****p*< 0.001.


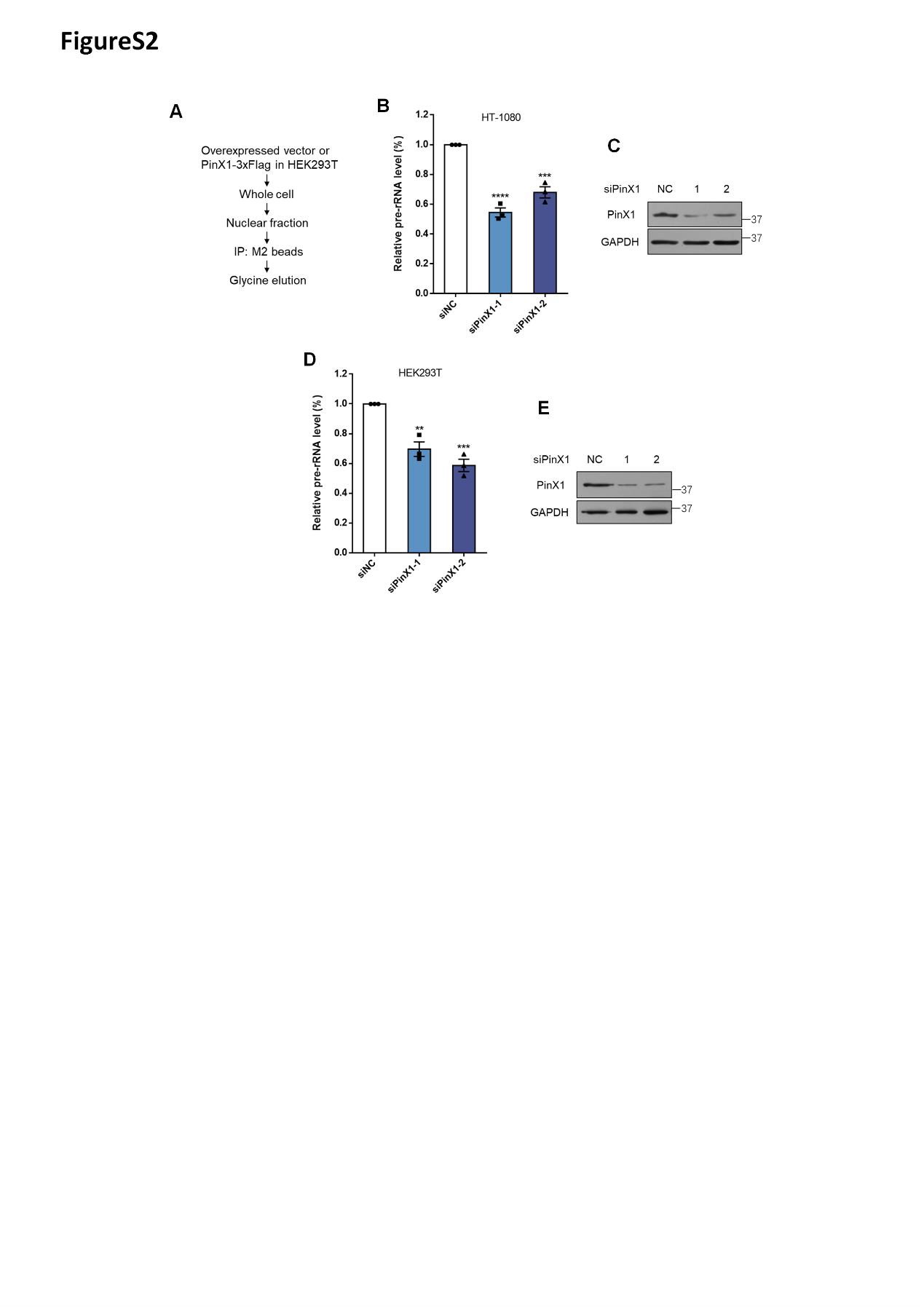


**Fig.s2 PinX1 is required for rDNA transcription**

(A) The schematic depicts the strategy of PinX1-interacted protein identification. (B) Pre-rRNA levels were determined by RT-qPCR in PinX1 wild type or PinX1 knockdown HT-1080 cells. (C) Validation of the knockdown efficiency of PinX1 in HT-1080 transfected with PinX1-specific short interfering RNAs (siPinX1) or control siRNA (siNC) using western blotting. (D) Pre-rRNA levels were determined by RT-qPCR in PinX1 wild type or PinX1 knockdown HEK293T cells. (E) Validation of the knockdown efficiency of PinX1 in HEK293T. All the error bars indicate the mean ± SD (n = 3, biological replicates). ***p*< 0.01, ****p*< 0.001, *****p*< 0.0001.


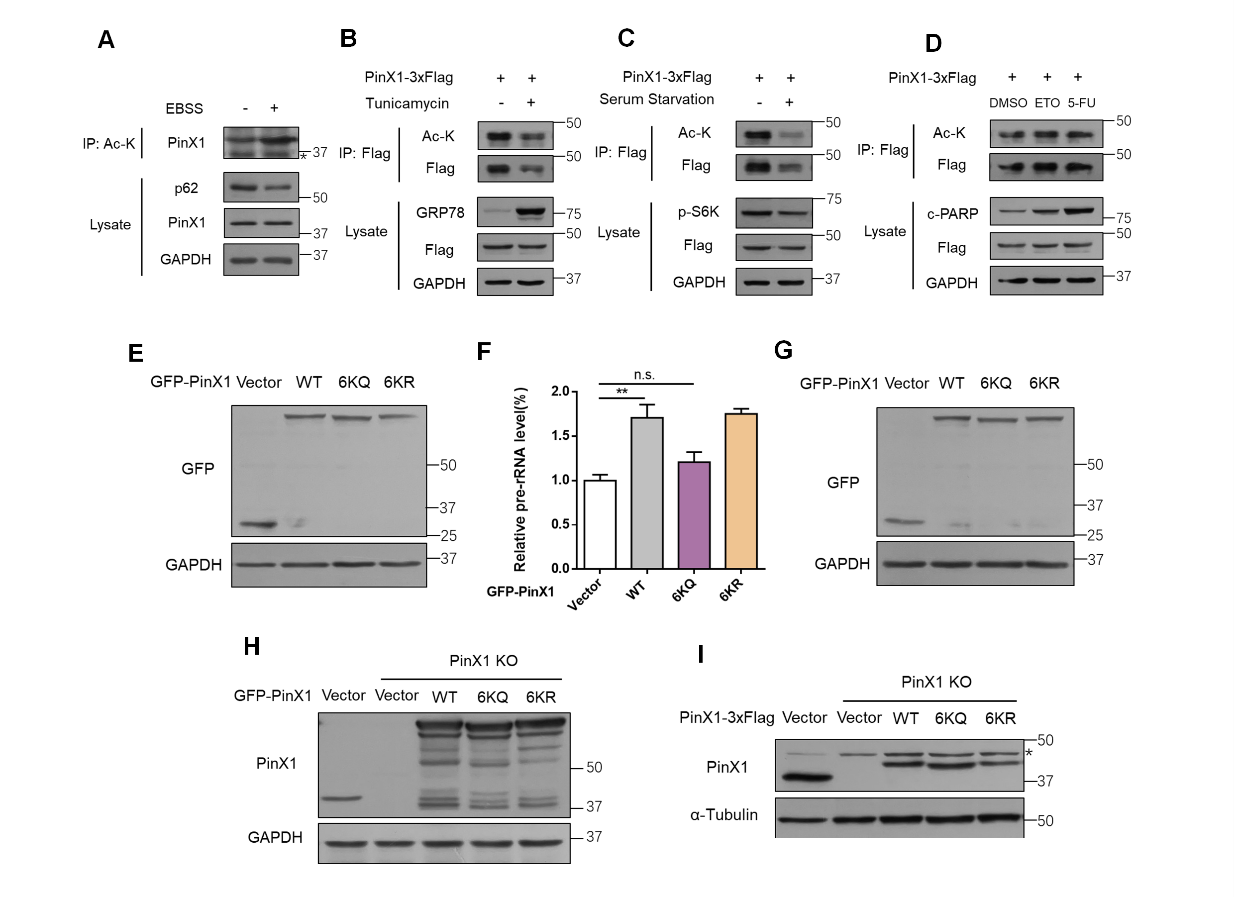


**Fig.s3 Acetylation levels of PinX1 do not alter under the endoplasmic reticulum stress, serum starvation or DNA damage stress.**

(A) Acetylation of endogenous PinX1 was determined in HCT116 with EBSS treated or not. Acetylated proteins were immunoprecipitated from cell lysate using acetyl lysine antibody affinity resin and endogenous PinX1 was detected by western blot. (B, C and D) HCT116 cells transfected with PinX1-3×Flag plasmid were treated with serum free DMEM, tunicamycin (1 μg/mL), etoposide (20 μM) and 5-fluorouracil (100 μM). After 24 h, the cells were harvested. Whole cell extracts were immunoprecipitated with an anti-Flag antibody affinity resin. Acetylation of PinX1 was detected by pan-acetyl-lysine antibody. (E) Western blotting analysis of GFP fusion proteins in transfected HT-1080 cells, related to Figure 3J. (F) Pre-rRNA levels were determined by RT-qPCR in HEK293T cells transiently expressing GFP-PinX1 WT, 6KR, 6KQ. Error bars indicate the mean ± SD (n = 3, biological replicates). **p*< 0.05, n.s., not significant. (G) Western blotting analysis of GFP fusion proteins in transfected HEK293T cells. (H) Western blotting was performed on the cell lysates for evaluating the indicated proteins in rescued HCT116 cell lines, related to Figure 3K. (I) Western blotting analysis of PinX1 in PinX1-KO and stable-rescued HCT116 cell lines, related to Figure 3L. The asterisk points to a nonspecific band.


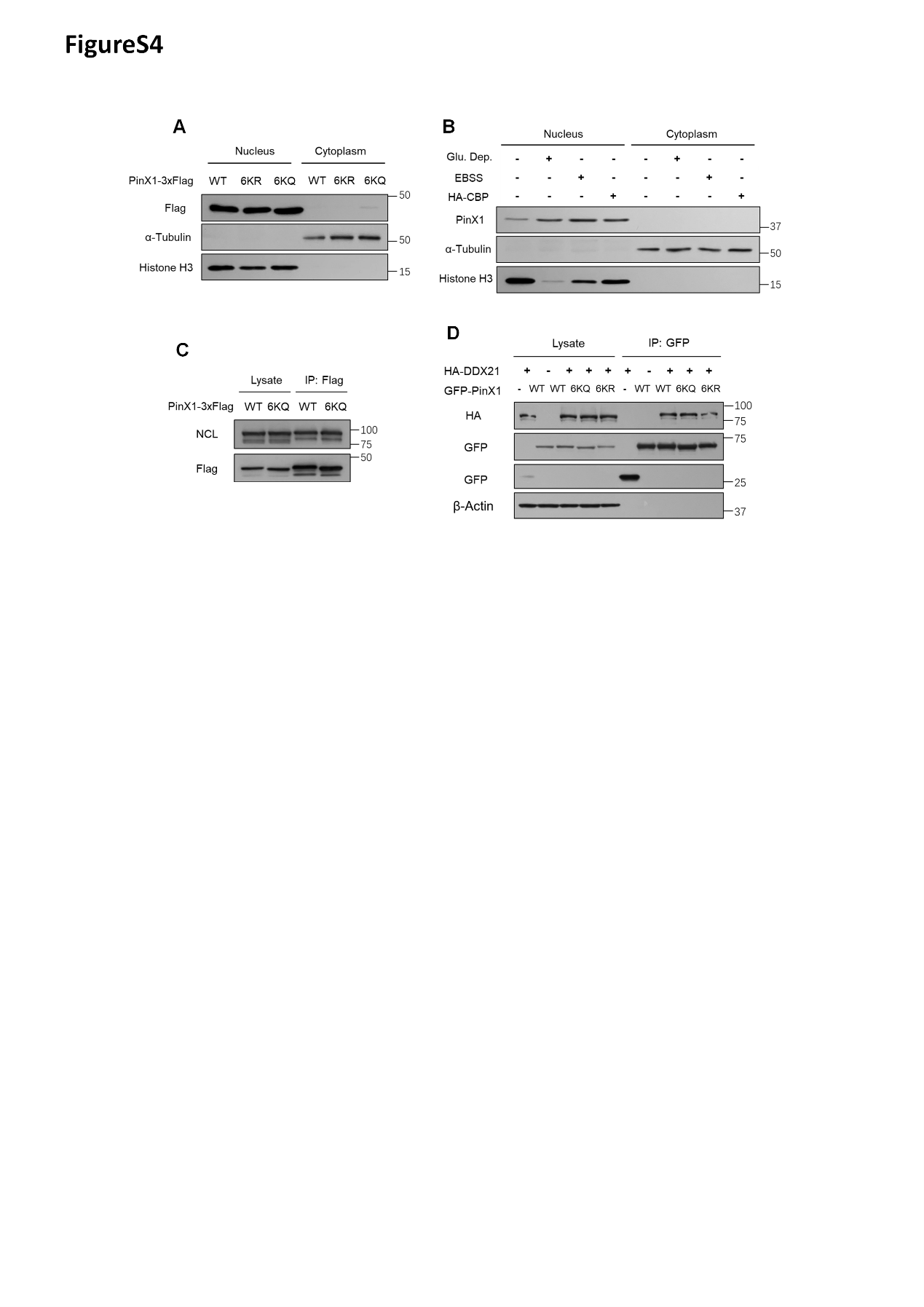


**Fig.s4 Acetylation of PinX1 affects neither its nuclear cytoplasmic translocation nor interaction with rRNA-processing proteins.**

(A) HCT116 cells were transfected with 3×Flag tagged PinX1 WT, 6KR or 6KQ mutant plasmids. Nuclear and cytoplasmic fraction separation experiments were performed to detect the localization of exogenous PinX1. (B) HCT116 cells were treated as indicated. Nuclear and cytoplasmic fraction separation experiments were performed to detect the localization of endogenous PinX1. (C) HEK293T cells were transfected with 3×Flag tagged PinX1 WT or 6KQ plasmids, and the protein was extracted for co-immunoprecipitation to detect the interaction with endogenous NCL. (D) HEK293T cells were transfected as indicated, and the protein was extracted for co-immunoprecipitation to detect the interaction with DDX21.


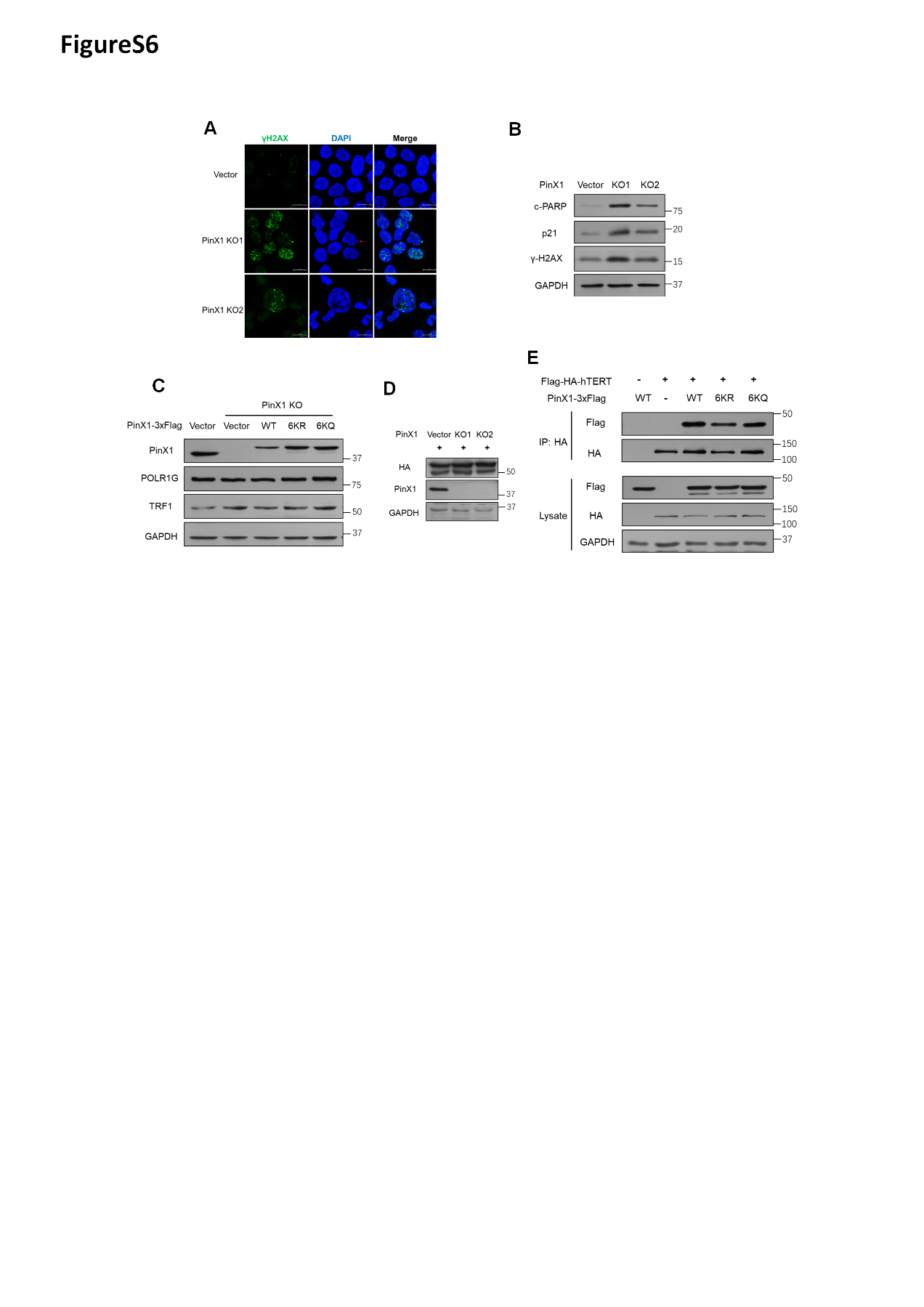


**Fig.s5 Acetylation of PinX1 does not regulate its classical targets TRF1 or hTERT.**

(A) Wild type and PinX1 knockout unsynchronized HCT116 cells were fixed, stained with DAPI (blue) to identify nuclei and micronuclei, and treated with antibodies to histone γ-H2AX (green) to detect damaged DNA. The red arrow points to a micronucleus that is positive for damaged DNA. Scale bar = 10 μm. (B) Wild type and PinX1 knockout HCT116 cells were subjected to Western blotting. (C) Wild type and PinX1 knockout HCT116 cells transiently transfected with empty vector, PinX1 WT, 6KR or 6KQ plasmids for 48 h were harvested and subjected to Western blotting. (D) HA tagged TRF1 plasmids were transfected into wild type or PinX1 knockout HCT116 cells. After 48 h, the cells were harvested and subjected to Western blotting. (E) HEK293T cells were transfected as indicated, and the protein was extracted for co-IP to detect the interaction between PinX1 and TERT.


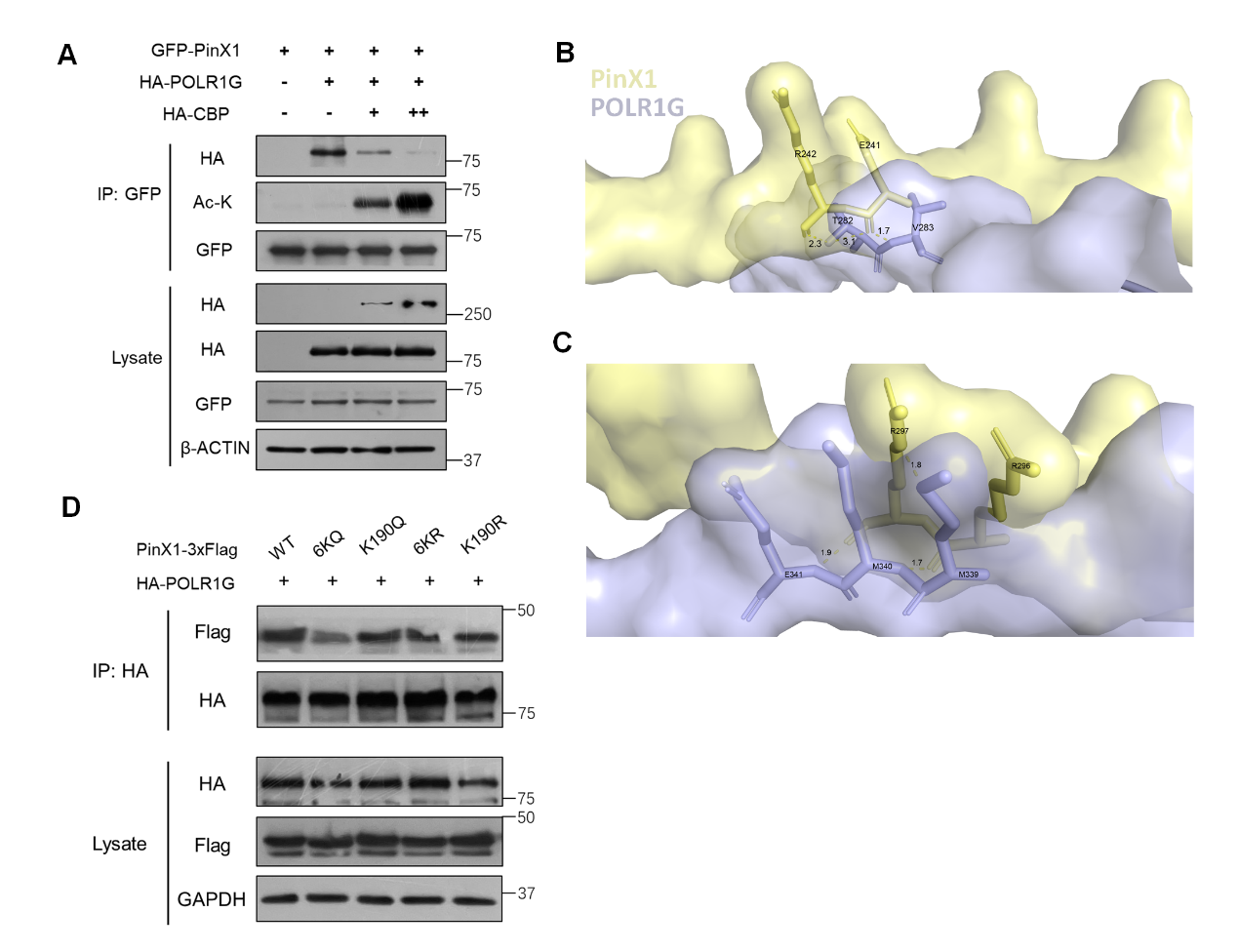


**Fig.s6 PinX1 interacts POLR1G through its C terminal.**

(A) GFP-tagged PinX1 and HA-POLR1G were co-transfected with different amount of HA-tagged CBP into HEK293T cells. The protein was extracted for co-immunoprecipitation to detect the interaction of PinX1 and POLR1G. (B and C) The predicted structure of human PinX1 and POLR1G from the AlphaFold2 database and the interacting interface was visualized in PyMOL. (D) WT, 6KQ, K190Q, 6KQ or K190R of PinX1 was transfected into HA-POLR1G expressed HCT116 and co-IP assay was used to detect the interaction of PinX1 and POLR1G.


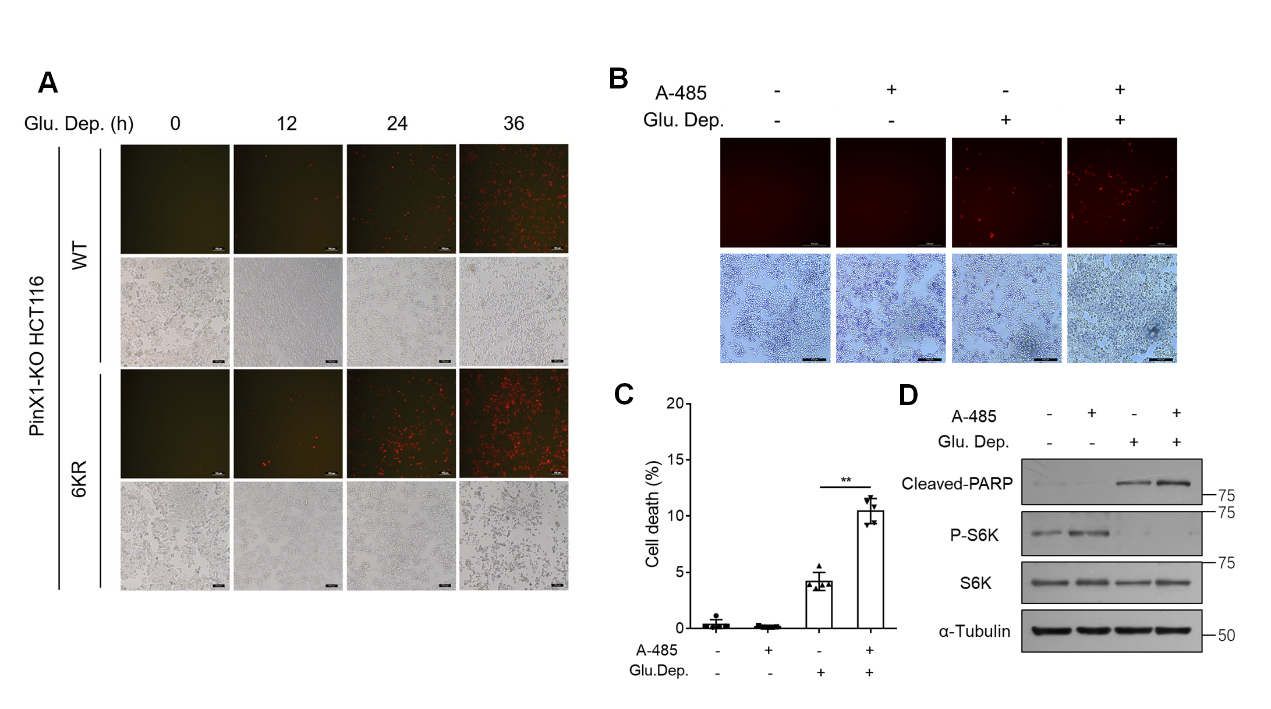


**Fig.s7 PinX1 acetylation promotes cell survival under glucose deprivation.**

(A) PinX1 WT or 6KR rescued HCT116 cells were cultured in glucose free medium for the indicated time (hours), followed by cell viability analyses using propidium iodide (PI) uptake assay, related to Figure 7G. Scale bars = 100 μm. (B) Cell viability was analyzed after A-485 (1 μM) pre-treatment for 24 hours and glucose deprivation treatment for 24 hours. (C) The percentage of cells positively stained with PI was quantified, related to (B). Data are shown as mean ± SD (n = 5), with ***p* < 0.01. (D) The levels of proteins were determined by Western blotting as indicated.
